# Hypothermia for Cardioprotection in Patients with St-Elevation Myocardial Infarction: Do Not Give It the Cold Shoulder Yet!

**DOI:** 10.3390/jcm11041082

**Published:** 2022-02-18

**Authors:** Mohamed El Farissi, Thomas P. Mast, Mileen R. D. van de Kar, Daimy M. M. Dillen, Jesse P. A. Demandt, Fabienne E. Vervaat, Rob Eerdekens, Simon A. G. Dello, Danielle C. Keulards, Jo M. Zelis, Marcel van ‘t Veer, Frederik M. Zimmermann, Nico H. J. Pijls, Luuk C. Otterspoor

**Affiliations:** 1 Department of Cardiology, Catharina Hospital, 5623 EJ Eindhoven, The Netherlands; thomas.mast@catharinaziekenhuis.nl (T.P.M.); mileen.vd.kar@catharinaziekenhuis.nl (M.R.D.v.d.K.); daimy.dillen@catharinaziekenhuis.nl (D.M.M.D.); jesse.demandt@catharinaziekenhuis.nl (J.P.A.D.); fabienne.vervaat@catharinaziekenhuis.nl (F.E.V.); rob.eerdekens@catharinaziekenhuis.nl (R.E.); s.dello@antoniusziekenhuis.nl (S.A.G.D.); danielle.keulards@catharinaziekenhuis.nl (D.C.K.); jo.zelis@catharinaziekenhuis.nl (J.M.Z.); marcel.vh.veer@catharinaziekenhuis.nl (M.v.‘t.V.); frederik.zimmermann@catharinaziekenhuis.nl (F.M.Z.); nico.pijls@xs4all.nl (N.H.J.P.); luuk.otterspoor@catharinaziekenhuis.nl (L.C.O.); 2 Department of Biomedical Engineering, Eindhoven University of Technology, 5612 AZ Eindhoven, The Netherlands

**Keywords:** acute myocardial infarction, STEMI, PCI, hypothermia, myocardial reperfusion injury

## Abstract

The timely revascularization of an occluded coronary artery is the cornerstone of treatment in patients with ST-elevation myocardial infarction (STEMI). As essential as this treatment is, it can also cause additional damage to cardiomyocytes that were still viable before reperfusion, increasing infarct size. This has been termed “myocardial reperfusion injury”. To date, there is still no effective treatment for myocardial reperfusion injury in patients with STEMI. While numerous attempts have been made to overcome this hurdle with various experimental therapies, the common denominator of these therapies is that, although they often work in the preclinical setting, they fail to demonstrate the same results in human trials. Hypothermia is an example of such a therapy. Although promising results were derived from experimental studies, multiple randomized controlled trials failed to do the same. This review includes a discussion of hypothermia as a potential treatment for myocardial reperfusion injury, including lessons learned from previous (negative) trials, advanced techniques and materials in current hypothermic treatment, and the possible future of hypothermia for cardioprotection in patients with STEMI.

## 1. Introduction

Coronary heart disease remains the leading cause of death worldwide, accounting for approximately >9 million deaths each year [1]. A large part of this mortality is directly related to acute myocardial infarction (AMI) [2].

In patients with ST-elevation myocardial infarction (STEMI), primary percutaneous coronary intervention (PPCI) is currently the treatment of choice to restore blood flow to the ischemic myocardium and to limit infarct size (IS) [3]. This decrease in IS, in turn, translates to improved clinical outcomes, such as heart failure and risks of mortality [4]. As essential as this treatment is, it can also cause additional damage to cardiomyocytes that were still viable until just before reperfusion [5]. This has been termed *myocardial reperfusion injury* [5]. Reperfusion triggers an inevitable inflammatory response (intended to promote healing but exceeding its goal) leading to additional injury. This may undo a considerable part of the recovery of the ischemic myocardium achieved by PPCI. Of note and important for the development of new therapies, the major part of this reperfusion injury occurs within the first few minutes after reperfusion [5].

To date, there is no effective treatment for myocardial reperfusion injury in patients with STEMI [5]. A major challenge is the translation of positive preclinical studies with experimental cardioprotective therapies to the clinical setting of patients with AMI. Hypothermia is a perfect example of such a therapy, as it entails multiple anti-inflammatory properties.

In other organs, for example in ischemia–reperfusion brain injury after cardiac arrest, therapeutic hypothermia has already been proven effective [6,7]. However, conflicting evidence of hypothermia regarding efficacy between preclinical studies and randomized controlled trials in patients with STEMI has led to a slight loss of confidence in hypothermia as a potential cardioprotective therapy. Still, should an age-old therapy such as hypothermia not enjoy more trust before throwing in the towel?

This review discusses hypothermia as a potential treatment for myocardial reperfusion injury, including lessons learned from previous (negative) trials, new developments in techniques and materials used in current day hypothermia treatment, and the possible future of hypothermia for cardioprotection in patients with STEMI.

## 2. Pathophysiology and Clinical Expression of Reperfusion Injury

When considering the pathophysiology of myocardial reperfusion injury, ischemia cannot go unmentioned, since most of the injurious mechanisms acting during reperfusion also play a role in counterbalancing the effects of ischemia. 

During ischemia, the oxygen-depleted cardiomyocytes will switch to anaerobic glycolysis, resulting in ATP depletion and the accumulation of lactate [8]. Consequently, a decrease in the intracellular pH to <7.0 activates the Na^+^–H^+^ ion exchanger, extruding protons from the cell in exchange for sodium [8]. The hypernatriemic state of the cardiomyocyte is enhanced through dysfunction of the Na^+^–K^+^ ATPase [9]. In order to eliminate the sodium ions, the reverse function of the Na^+^–Ca^2+^ ion exchanger, together with a decrease in active Ca^2+^ efflux due to ATP depletion, will result in the accumulation of intracellular Ca^2+^ [8,9]. 

By halting the oxidative phosphorylation, the mitochondrial membrane depolarizes [10]. In order to maintain mitochondrial membrane potential, any available ATP will be utilized by the reverse function of F_1_F_0_ ATPase, resulting in a rapid fall in ATP levels after the onset of ischemia [10]. 

A key feature of myocardial reperfusion injury is the opening of the mitochondrial permeability transition pore (MPTP) [11]. The loss of the inhibitory effects on MPTP opening results from a quick restoration of the intracellular pH (after reperfusion) by driving out protons, while the recovered 3Na^+^–2K^+^ ATPase restores the intracellular sodium concentration [11]. The overload of intracellular calcium significantly increases after reperfusion through reactive oxygen species (ROS)-induced damage to the sarcoplasmic reticulum. As such, calcium also accumulates in the mitochondria via the mitochondrial Ca2+ uniporter. This also results in MPTP opening [10,11,12]. The opening of the MPTP depolarizes the mitochondrial membrane. This uncouples oxidative phosphorylation and subsequently results in progressive ATP depletion [10,11,12]. 

Due to the supraphysiological levels of intracellular calcium and the earlier-mentioned restoration of the pH, a state of hypercontracture of the myofibrils can be observed [13,14]. The latter, in combination with a state of ATP depletion, results in the cell death of the myocytes that initially survived ischemia, a process known as “lethal myocardial reperfusion injury” [5]. This will result in an increase in IS. 

Clinically, myocardial reperfusion injury consists of four features, i.e., myocardial stunning, reperfusion-induced arrhythmias, the no-reflow phenomenon, and the earlier-mentioned lethal myocardial reperfusion injury [5]. Since these undesirable phenomena mostly occur in the first minutes after reperfusion, it seems that they are the result of reperfusion. Hypothermia affects multiple signaling pathways (e.g., Akt and SUMO) involved in myocardial reperfusion injury, ultimately preserving mitochondrial function and preventing the hypercontractile state of myofibrils [15,16,17]. 

## 3. Preclinical Studies

Preclinical data on cardioprotection in general have been obtained in different species and using different study protocols. As a result, there are still unanswered questions that need to be elucidated in order to improve cardioprotection for STEMI patients. A need for standardized methods has become evident in the past years, and consensus guidelines were only proposed recently [18]. 

Hypothermia for cardioprotection in AMI has also been investigated extensively [19,20,21,22]. It has become evident that hypothermia should be initiated as soon as possible, but most definitely before reperfusion. If hypothermia is initiated only after reperfusion (delayed treatment), it still exerts some effects on the microvasculature. Hypothermia decreases microvascular obstruction (MVO), an important feature of myocardial reperfusion injury [23,24].

Local hypothermia for AMI is of special interest to the authors. It has many advantages over systemic hypothermia for cardioprotection in patients with STEMI. The most practical way to achieve local hypothermia is by intracoronary saline infusion. 

In 2005, Kim et al. [25] evaluated the feasibility and safety of intracoronary hypothermia through the infusion of lactated Ringer’s solution (at room temperature and at 15 °C), distal to a ligated left anterior descending (LAD) artery in open-chest pigs. There was a linear relationship between the infusion solution temperature and infusion rate versus myocardial temperature with a temperature gradient of −2 °C between the distal LAD artery and the corresponding MaR, respectively. Otake et al. [20] evaluated intracoronary hypothermia through saline infusion in pigs. When initiated during ischemia (before reperfusion), intracoronary hypothermia significantly decreases IS/MaR compared to saline infusion at 36.5 °C (respectively 9 ± 2% vs. 36 ± 4%, *p* < 0.0001). This effect was less evident when intracoronary hypothermia was initiated after reperfusion in comparison to normal reperfusion (respectively 33 ± 2% vs. 45 ± 5%, *p* = 0.08).

For hypothermia to be effective as a cardioprotective therapy and decrease IS, the lessons learned from these preclinical studies should be taken into consideration when organizing a randomized controlled trial. While it is clear from these preclinical studies that hypothermia should be initiated before reperfusion, it is still undesirable to delay reperfusion for too long, because we know all too well that “time equals muscle”. What, then, are the minimum requirements for successful hypothermic cardioprotection?

## 4. Clinical Studies

**Figure 1:**
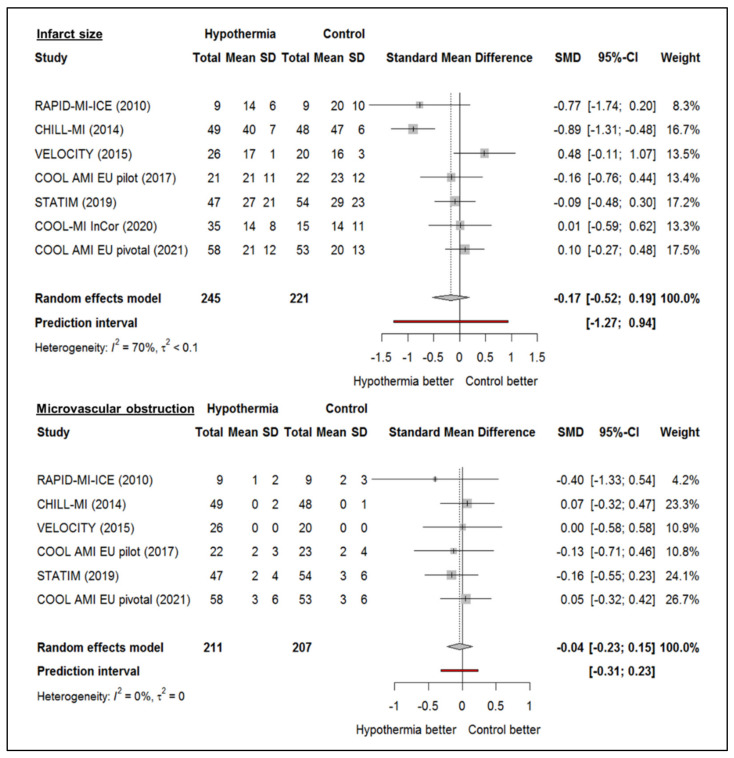
Study-level meta-analysis of randomized trials investigating systemic hypothermia for cardioprotection in STEMI and effect on infarct size and microvascular obstruction [26,27,28,29,30,31,32].

**Figure 2:**
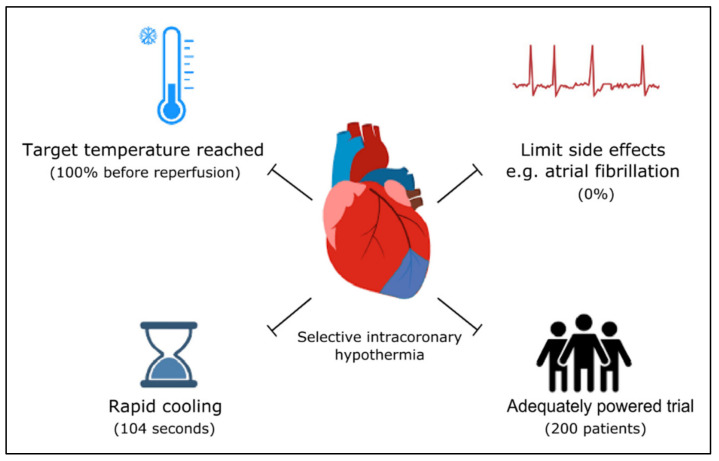
Important requirements for successful hypothermic cardioprotection in patients with ST-elevation myocardial infarction. In parentheses are the numbers related to selective intracoronary hypothermia from the ongoing EURO-ICE study.

Multiple randomized controlled trials have studied hypothermia for cardioprotection, and all were negative for the primary endpoint, either IS or IS/MaR [26,27,28,29,30,31,32]. A study-level meta-analysis of randomized trials investigating systemic hypothermia for cardioprotection in STEMI (Figure 1) did not show a decrease in IS compared to standard PCI (standardized mean difference of IS −0.17, 95% confidence interval −0.52 to 0.19). Specifically, MVO was also unaffected by systemic hypothermia (standardized mean difference of MVO −0.04, 95% confidence interval −0.23 to 0.15). A critical appraisal of these studies revealed a number of important limitations that may have contributed to these negative outcomes (Figure 2).

First, systemic hypothermia is unable to cool fast enough, i.e., before reperfusion and without delaying reperfusion too long. It is obvious that cooling methods that intend to cool the entire body are by definition time-consuming. Even the strongest cooling devices cause a delay in treatment [32]. This delay (with longer ischemic time) is at the expense of the benefit achieved by hypothermia. Second, the target temperature was not reached before reperfusion, or not at all in a number of patients in these trials [33]. This limitation is closely related to the previous. Third, systemic hypothermia causes side effects. Shivering occurred in all patients, necessitating the need for anti-shivering medication [26,27,28,29,30,31,32]. Furthermore, systemic hypothermia may provoke atrial arrhythmias [32]. Fourth, all studies investigating systemic hypothermia included patients without knowing their Thrombolysis in Myocardial Infarction (TIMI) grade flow, since it was necessary to start cooling before the angiogram was performed. Therefore, up to 30% of AMI patients were included in whom reperfusion (and therefore reperfusion injury) had already occurred [33]. Since hypothermia only exerts cardioprotection when initiated before reperfusion, a potential effect of hypothermia in these patients could not be expected. Final, wrong assumptions were frequently used for the calculation of study power. Due to an overestimation of the presumed effect of hypothermia on IS, too few patients were enrolled to demonstrate a significant decrease in IS [33]. In addition, in several studies, patients with only moderate IS were also included, making it more difficult to find statistically significant benefits of cooling. Conceptually, in a proof-of-concept study, only patients with large AMI should be included [18].

These limitations may explain why randomized controlled trials do not support the use of systemic hypothermia as a means of cardioprotection in patients with AMI. To turn the tide for hypothermic cardioprotection, in STEMI patients, sophisticated solutions for the limitation of systemic hypothermia should continue to be developed.

## 5. Improving Hypothermic Cardioprotection

In order to develop a cooling technique that is so fast that all patients can be cooled sufficiently and before reperfusion, the most logical step is through local cooling. From a practical point of view, local cooling of the MaR can be achieved rapidly by using the coronary arteries as an access to the corresponding myocardium. This creates opportunities for the direct cooling of the region, and would be much faster than current-day cooling techniques. The unnecessary cooling of a healthy myocardium is avoided in this manner. Side effects are also less likely with such local cooling. 

In order to prove the effect of hypothermia as a cardioprotective therapy, randomized controlled trials should only include patients with TIMI grade flow 0 or 1 [18]. Besides this, larger sample sizes should be used, as the presumed effect may be smaller than previously thought. In this context, selective intracoronary hypothermia cannot go unmentioned.

## 6. Selective Intracoronary Hypothermia

Recently, a new method of selective intracoronary hypothermia was developed, intended to reduce myocardial reperfusion injury and decrease IS. First, the occlusion is crossed with a regular guidewire. Thereafter, an over-the-wire balloon (OTWB) is advanced over the guidewire and inflated at the site of the occlusion. Next, a pressure/temperature wire (PressureWire™ X; Abbott, St. Paul, MN, USA) is advanced into the distal coronary artery for the continuous recording of pressure and temperature. It may be necessary to deflate the OTWB for a short period of time to get the pressure/temperature wire past the balloon. After the guidewire is removed from the central lumen of the OTWB, this lumen is connected to two infusion pumps filled with saline at room temperature and 4 °C, respectively. First, saline at room temperature is infused for 7–10 min at a flow rate of 15–30 mL/min (occlusion phase) to maintain a distal coronary temperature of 6–8 °C below body temperature. Next, the OTWB is deflated and the infusion continues for 7–10 more minutes, using the second infusion pump filled with saline at 4 °C (reperfusion phase). The flow rate can be varied to maintain a distal coronary temperature of between 4 and 6 °C below body temperature. Finally, the OTWB is retracted and the PressureWire X can be used as a guidewire for the placement of a stent. 

**Figure 3:**
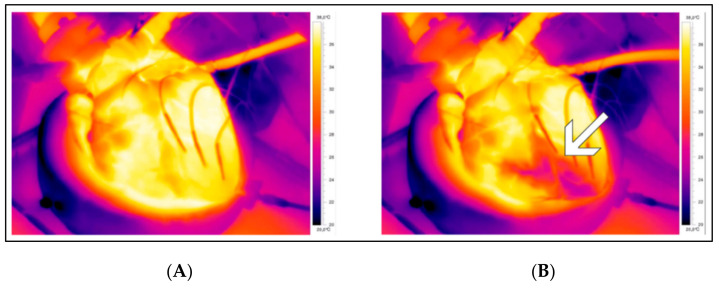
Visualization of the infarct area before (**A**) and during (**B**) selective intracoronary hypothermia in an ex vivo beating porcine heart. The arrow indicates the myocardium at risk (MaR) during selective intracoronary hypothermia. The decrease in myocardial temperature is limited to the borders of the MaR without affecting the temperature of the healthy adjacent myocardium.

The relationship between intracoronary saline infusion distal to an occlusion, the distal intracoronary temperature and the temperature of the corresponding MaR in isolated beating porcine hearts during treatment with selective intracoronary hypothermia was investigated (Figure 3) [34]. A temperature gradient existed during the occlusion phase, and no relevant temperature gradient was present in the reperfusion phase [34]. 

## 7. Ongoing Studies

A safety and feasibility study investigating selective intracoronary hypothermia in 10 patients with STEMI demonstrated symptomatic atrioventricular conduction disturbances in 2 of 4 patients with inferior STEMI and an occluded right coronary artery [35]. In six patients with large anterior STEMI, no complications were observed [35]. 

Therefore, in the ongoing prospective randomized controlled EURO-ICE trial (European Intracoronary Cooling Evaluation in Patients With ST-Elevation Myocardial Infarction; Clinicaltrials.gov identifier: NCT03447834), only patients with anterior STEMI are being recruited [36]. This is the largest hypothermia trial in patients with STEMI so far. A predetermined safety analysis did not show any safety concerns in the first 50 patients treated with selective intracoronary hypothermia during PPCI in comparison with standard PPCI [37].

Interestingly, and in contrast to studies with systemic hypothermia, atrial fibrillation did not occur in the intracoronary hypothermia group, compared with three patients in the control group [37].

Definite evidence as to whether selective intracoronary hypothermia can reduce IS will follow after the completion of the EURO-ICE trial. Ultimately, only a beneficial impact on hard clinical endpoints at follow-up, such as heart failure and mortality, may lead to the implementation of selective intracoronary hypothermia into the STEMI-PPCI pathway.

## 8. The Future of Therapeutic Hypothermia in STEMI

Although we believe that selective intracoronary hypothermia is the logical next step in the evolution of therapeutic hypothermia in AMI during primary PCI, we are aware of the uncertainties that still remain. The duration and depth of cooling are still unclear, and this obviously needs clarification to make hypothermia more effective. To the best of our knowledge, dose–response studies are yet to be performed. If a prolonged duration of hypothermia seems necessary, additional methods beyond the procedure of primary PCI itself should be developed and studied. This can be accomplished by prolonging cooling itself or by adding pharmacological stimuli to induce hypothermia. An example is cholecystokinin octapeptide (CCK8), which has the ability to induce hypothermia when injected peripherally [38,39]. *Hypothermia in syringe*, through activation of the same cell signaling pathways that are activated during cooling, should mediate similar effects without decreasing the actual body temperature. In other words, cooling is not the main objective in this regard, but rather recreating the same signaling environment as during cold stress [39]. However, this is still very speculative. 

We should also consider combining hypothermia with other cardioprotective therapies, such as the administration of supersaturated oxygen therapy (easy to combine with intracoronary saline infusion) or left ventricular unloading before reperfusion. This may enhance its cardioprotective effects. These therapies are also discussed in this special issue, *Interventional Cardiology: Current Challenges in Acute Myocardial Infarction* [40]. Studies on combinations of such therapies are necessary in order to clarify this position.

## 9. Conclusions

Therapeutic hypothermia, although hampered by conflicting results between preclinical studies and negative randomized controlled trials so far, remains a promising cardioprotective treatment for patients with STEMI. The prerequisites for the successful translation of hypothermia include continuous innovations to overcome the well-known limitations of systemic hypothermia. Ultimately, if hypothermia can decrease infarct size and improve clinical outcome in patients with STEMI, this will be the next step beyond PPCI. 

## Abbreviations

AMI	Acute myocardial infarction
STEMI	ST-elevation myocardial infarction
PCI	Percutaneous coronary intervention
IS	Infarct size
MaR	Myocardium at risk
MVO	Microvascular obstruction
